# Differential Antioxidant Responses and Perturbed Porphyrin Biosynthesis after Exposure to Oxyfluorfen and Methyl Viologen in *Oryza sativa*

**DOI:** 10.3390/ijms160716529

**Published:** 2015-07-21

**Authors:** Nhi-Thi Pham, Jin-Gil Kim, Sunyo Jung

**Affiliations:** School of Life Sciences and Biotechnology, BK21 Plus KNU Creative Bioresearch Group, Kyungpook National University, Daegu 702-701, Korea; E-Mails: nhipt.hus@gmail.com (N.-T.P.); truewaykor@naver.com (J.-G.K.)

**Keywords:** antioxidant, methyl viologen, oxyfluorfen, photooxidative stress, porphyrin

## Abstract

We compared antioxidant responses and regulation of porphyrin metabolism in rice plants treated with oxyfluorfen (OF) or methyl viologen (MV). Plants treated with MV exhibited not only greater increases in conductivity and malondialdehyde but also a greater decline in F_v_/F_m_, compared to plants treated with OF. MV-treated plants had greater increases in activities of superoxide dismutase (SOD) and catalase (CAT) as well as transcript levels of *SODA* and *CATA* than OF-treated plants after 28 h of the treatments, whereas increases in ascorbate peroxidase (APX) activity and transcript levels of *APXA* and *APXB* were greater in OF-treated plants. Both OF- and MV-treated plants resulted in not only down-regulation of most genes involved in porphyrin biosynthesis but also disappearance of Mg-porphyrins during the late stage of photooxidative stress. By contrast, up-regulation of heme oxygenase 2 (*HO2*) is possibly part of an efficient antioxidant response to compensate photooxidative damage in both treatments. Our data show that down-regulated biosynthesis and degradation dynamics of porphyrin intermediates have important roles in photoprotection of plants from perturbed porphyrin biosynthesis and photosynthetic electron transport. This study suggests that porphyrin scavenging as well as strong antioxidative activities are required for mitigating reactive oxygen species (ROS) production under photooxidative stress caused by OF and MV.

## 1. Introduction

The porphyrin biosynthetic pathway provides the vital cofactors and pigments for chlorophylls, heme, siroheme, and phytochromobilin. The biosynthesis of porphyrin in all living cells occurs through several steps where the formation of 5-aminolevulinic acid (ALA) is the first committed intermediate [1] (Supplementary Figure S1). Protoporphyrinogen oxidase (PPO), which catalyzes the oxidation of protoporphyrinogen IX (Protogen IX) to protoporphyrin IX (Proto IX), is the last enzyme before the branch in the porphyrin biosynthetic pathway, and its product, Proto IX, is directed to the magnesium (Mg) and iron (Fe) branches for chlorophyll and heme biosynthesis, respectively [1,2]. Many intermediates in the porphyrin biosynthetic pathway, such as Proto IX and protochlorophyllide (Pchlide), interact with molecular oxygen in the presence of light to form reactive oxygen species (ROS), which is harmful to cells and causes the peroxidation of membrane lipids [3,4].

The inhibition of PPO activity by peroxidizing herbicide oxyfluorfen (OF) results in the accumulation of Protogen IX, which diffuses to the cytoplasm and is oxidized to Proto IX via peroxidase-like enzymes in membrane [5]. Cytoplasmic Proto IX is a potent photosensitizer resulting in the formation of singlet oxygen (^1^O_2_), causing cell death [5,6]. Methyl viologen (MV) also causes rapid membrane damage in plants, but the action mechanism is different from OF. MV interrupts photosynthetic electron transport (PET) by accepting electrons from photosystem I (PSI) and transferring them to molecular oxygen, thereby resulting in the production of toxic superoxide (O_2_·^−^), which efficiently induces cell death [7,8]. In particular, the reaction of hydroxy radicals with unsaturated lipids sets up a chain reaction that, once initiated, propagates lipid peroxidation [9]. Membrane damage can further release free forms of chlorophyll biosynthetic intermediates, which are a powerful photosensitizer and a generator of highly reactive ^1^O_2_ in the presence of light.

Porphyrin biosynthesis and degradation are carefully adjusted to the cellular requirements, reflecting the different needs under varying stress conditions including water stress and peroxidizing herbicide [3,10,11,12,13]. Under deregulated porphyrin biosynthesis, excited porphyrins tend to spread into other cellular compartments that are less well protected against their photodynamic action. The extent to which antioxidant responses can counteract the overproduction of ROS may determine whether or not plants can tolerate the stress [14]. The extent of stress-induced damage can be attenuated by the action of the cell’s antioxidant systems. The O_2_·^−^ disproportionates to oxygen and peroxide via the action of chloroplastic superoxide dismutase (SOD). Ascorbate peroxidase (APX), peroxidase (POD), and catalase (CAT) catalyze the conversion of H_2_O_2_ to water [14,15]. The elucidation of plant resistance to MV-induced oxidative stress has mainly been focused on antioxidant enzymes [16,17], photoprotective mechanism [18,19], and intracellular transport of MV [20,21] in previous studies. There is little known about differential regulatory mechanism of porphyrin biosynthesis and mitigation of ROS under oxidative stress induced by OF and MV.

In this study, we examined the activity and expression levels of antioxidant enzymes in rice plants under different types of photooxidative stress caused by the pro-oxidants, OF and MV. We also questioned how porphyrin metabolism is regulated to mitigate ROS production in the stressed plants. The plants treated with MV that inhibits PET showed differential changes in porphyrin metabolism as well as photooxidative damage and antioxidant responses, compared to the plants treated with OF that directly inhibits porphyrin biosynthesis. Our study suggests that porphyrin scavenging as well as strong antioxidative activities are demanded for alleviating the accumulation of ROS under photooxidative stress imposed by OF and MV.

## 2. Results and Discussion

### 2.1. Differential Photooxidative Stress Responses in Rice Plants Treated with Oxyfluorfen (OF) and Methyl Viologen (MV)

**Figure 1:**
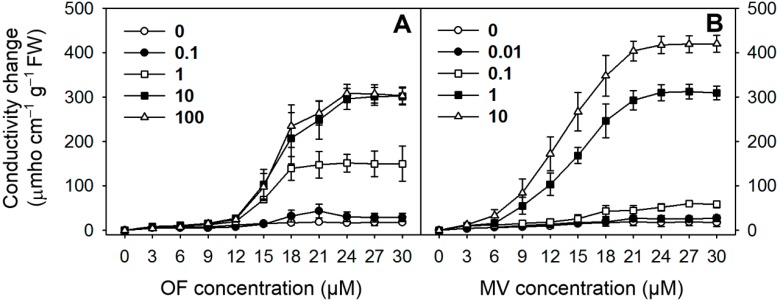
Effect of oxyfluorfen (OF) (**A**) and methyl viologen (MV) (**B**) on cellular leakage. Leaf segments were incubated with various concentrations of either OF or MV. Tissues were exposed to continuous white light at 250 µmol·m^−2^·s^−1^ photosynthetic photon flux density for 30 h following a 12-h dark incubation. Then conductivity, which reflects electrolyte leakage, was measured in the bathing solution for 30 h. Each symbol indicates the concentration of OF or MV.

Leaf disks were incubated with either OF or MV, which inhibits a key enzyme PPO at the branch point of porphyrin biosynthesis [5] or PET in PS I [7], respectively. The treated leaf disks exhibited increases in conductivity, an indicator of cellular leakage, after exposure to illumination following a 12-h dark incubation. The conductivity in OF-treated leaf tissues began to greatly increase 15 h after illumination at concentrations from 1 to 100 µM OF and kept almost constant 24 h after illumination (Figure 1A). The conductivity in MV-treated leaf tissues began to markedly increase 9 h after illumination at concentrations above 1 µM MV and kept constant 24 h after illumination (Figure 1B), demonstrating a faster increase in conductivity, compared to that of OF-treated leaf tissues.

**Figure 2:**
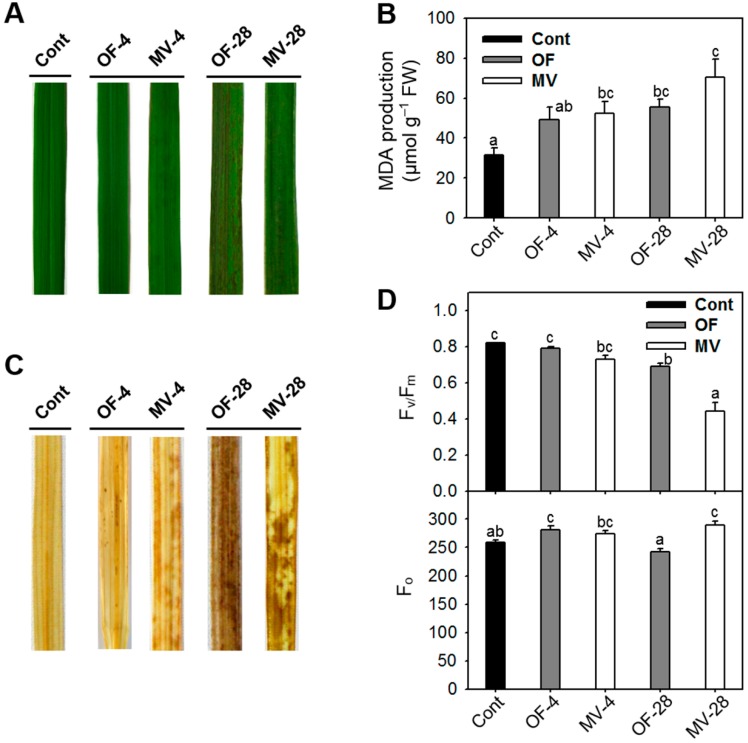
Necrotic symptoms and oxidative metabolism of rice plants with the foliar application of OF or MV treatment. (**A**) Necrotic symptoms on leaves. Three-week-old rice plants were sprayed with either 50 µM OF or 50 µM MV, placed in darkness for 12 h to allow absorbance, and then illuminated for either 6 or 30 h; (**B**) Malondialdehyde (MDA) content; (**C**) H_2_O_2_-3-diaminobenzidine (DAB)-staining. The production of H_2_O_2_ was visually detected by browning of leaf veins after DAB incubation; and (**D**) Photosynthetic performance. The efficiency of PSII photochemistry, F_v_/F_m_, was used to assess the functional damage to the plants. Cont, control; OF-4 and OF-28, four and 28 h after OF treatment, respectively; MV-4 and MV-28, four and 28 h after MV treatment, respectively. The data represent the mean ± SE of six replicates from two independent experiments. Means denoted by the same letter did not differ significantly at *p* < 0.05 according to LSD test. Different letters indicate significant difference in statistics.

To examine alterations in oxidative metabolism, rice plants were exposed to oxidative stress by foliar application of either OF or MV. Plants treated with 50 µM OF exhibited noticeable necrotic spots on leaves after 28 h of the treatment, whereas plants treated with 50 µM MV began to develop noticeable brown spots on leaves after 4 h of the treatment, with severe necrosis after 28 h (Figure 2A). Oxidative lipid damage is a marker of oxidative stress in plant tissues. Malondialdehyde (MDA) content, which is a measure of lipid peroxidation, began to increase in OF- and MV-treated plants after 4 h of the treatments, with further increase after 28 h of MV treatment (Figure 2B). Membrane disruption by nonenzymatic lipid peroxidation destroys cellular compartments, causes loss of solutes and dehydration, and finally leads to cell death [22]. To investigate whether these pro-oxidants influence ROS generation in the treated tissues, untreated leaves as well as OF- and MV-treated leaves were incubated with, 3-diaminobenzidine (DAB), which is a marker of H_2_O_2_ accumulation [23]. The H_2_O_2_ production was greater in MV-treated leaves than in OF-treated leaves after 4 h of the treatments, whereas OF-treated leaves exhibited more production of H_2_O_2_ than MV-treated leaves after 28 h (Figure 2B). Control leaves did not show any marked production of H_2_O_2_.

The effect of OF and MV on photosynthetic performance was verified by measuring changes in photochemical quantum efficiency, F_v_/F_m_, a trait positively correlated with the organization and vitality of photosystem II (PSII). The photooxidative stress caused by foliar application of OF and MV gradually decreased F_v_/F_m_ during the treatments, as compared to controls (Figure 2C). When exposed to light, excess porphyrins in OF-treated plants were photosensitized and led to produce ROS [5,6], which destroyed membrane lipids and decreased efficiency of PSII photochemistry through photodynamic reaction. MV blocks PET by accepting electrons from PSI, resulting in depletion of the reduced form of nicotinamide adenine dinucleotide phosphate (NADPH) inhibition of CO_2_ fixation, and significant damage to PSII activity [16,24]. The greater drop in F_v_/F_m_ after 28 h of MV treatment may result from a slight increase in F_o_ (Figure 2C) in addition to the inhibition of PET. The initial fluorescence F_o_, which is known to be affected by structural changes in the PSII complex [25], slightly increased after MV treatment, whereas it decreased in OF-treated plants (Figure 2C). The increased F_o_ level is probably associated not only with low PSII photochemistry but also low K_T_, which is the rate constant for energy transfer between antennae and reaction center and related to excitation energy transfer to non-fluorescent pigments [25], although the precise reasons for the decline remain to be determined. Overall, MV-treated plants displayed greater increases in cellular leakages and MDA production, markers of membrane disruption, as well as a substantial decline in F_v_/F_m_.

### 2.2. Effect of OF and MV on Activities and Expression Levels of Reactive Oxygen Species (ROS)-Scavenging Enzymes

**Figure 3:**
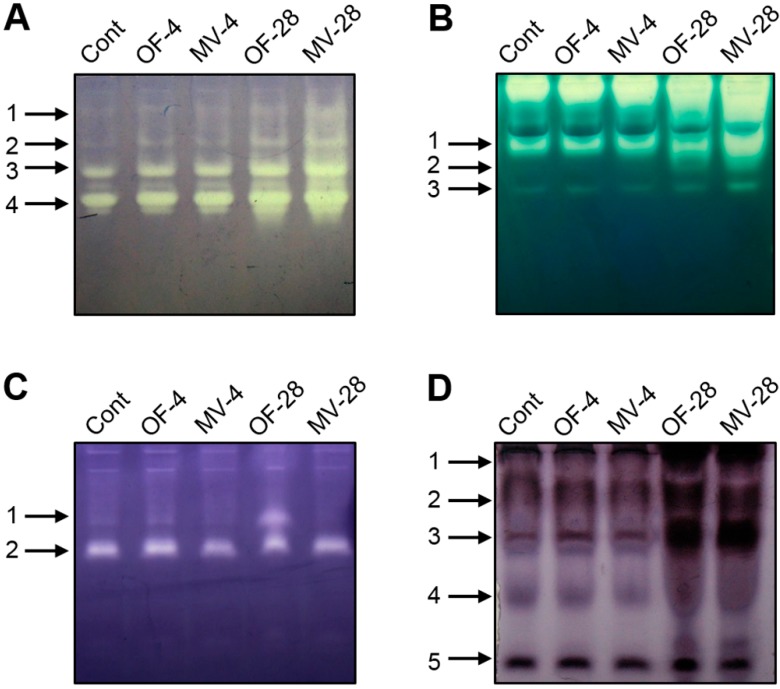
Profiles of antioxidant isozymes in rice plants with the foliar application of OF or MV treatment. (**A**) Superoxide dismutase; (**B**) Catalase; (**C**) Ascorbate peroxidase; and (**D**) Peroxidase. Non-denaturing activity gels were prepared and run as described in the method. The plants were subjected to the same treatments as in Figure 2. Treatment notations are the same as in Figure 2. The numbers indicate each isozyme of antioxidant enzymes in order of detected bands from the top.

The steady-state levels of H_2_O_2_, ^1^O_2_, O_2_·^−^, and the hydroxyl radical greatly depend on not only ROS-scavenging enzymes but also low molecular weight antioxidants including ascorbate, glutathione, and carotenoids in plant cells [15,26]. We monitored changes in isozyme profiles and transcript levels of ROS-scavenging enzymes in response to foliar application of OF and MV. The activities of all SOD isozymes were gradually increased during OF and MV treatment, with a greater increase after two days of MV treatment (Figure 3A). Oxidative stress induced by OF and MV led to a chain of further endogenous protective reactions. A set of H_2_O_2_-decomposing enzymes, namely CAT, APX, and POD [15,26], are heme-containing enzymes with Proto IX moieties [10,26]. The CAT isozymes 1, 2 and 3 increased after 28 h of OF and MV treatment, with a greater increase in MV-treated plants (Figure 3B). Staining activity of APX isozyme 1 increased only after 28 h of OF treatment, whereas APX isozyme 2 did not change during OF and MV treatment (Figure 3C). In comparison to untreated controls, staining activities of POD isozymes 1 and 3 prominently increased after 28 h of OF and MV treatments (Figure 3D). These results indicate an increased H_2_O_2_ quenching capacity by CAT, POD, and APX during stress responses induced by OF and MV.

**Figure 4:**
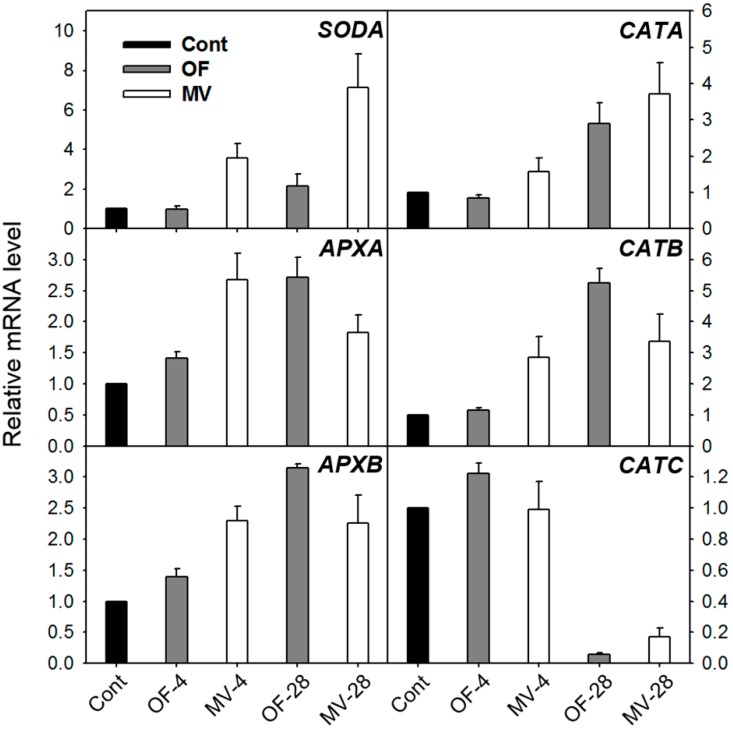
Expression of genes encoding the ROS-scavenging enzymes in rice plants with the foliar application of OF or MV treatment. Total RNAs were purified from plants and reverse transcribed. The resultant cDNAs were used as templates for qRT-PCR using *Actin* as an internal control. The control was used for normalization, with the expression level of the sample set to 1. Error bars represent SE, and representative data from three independent experiments are presented. The plants were subjected to the same treatments as in Figure 2. Treatment notations are the same as in Figure 2.

In parallel with the increased activities of ROS-scavenging enzymes, the OF and MV-treated plants responded to photooxidative stress by greatly up-regulating transcript levels of ROS-scavenging genes. The stronger increase in *SODA* transcript corresponded well to the greater SOD activity after 28 h of MV treatment, compared to the OF-treated plants (Figure 3 and Figure 4). Transcript levels of *APXA* and *APXB* gradually increased during OF treatment, whereas they reached the highest levels after 4 h of MV treatment. The OF- and MV-treated plants began to increase transcript levels of *CATA* and *CATB* 28 h and 4 h after the treatment, respectively. Transcript level of *CATC* increased slightly after 4 h of OF treatment, but decreased greatly after 28 h of OF and MV treatments. In our study, the increased activities of ROS-scavenging enzymes in OF- and MV-treated plants greatly enhanced the capacity to eliminate adverse impacts of ROS; however, this was not sufficient for avoiding photooxidative damage.

### 2.3. Photooxidative Stress-Induced Changes in Porphyrin Intermediates and Their Biosynthetic Genes

**Figure 5:**
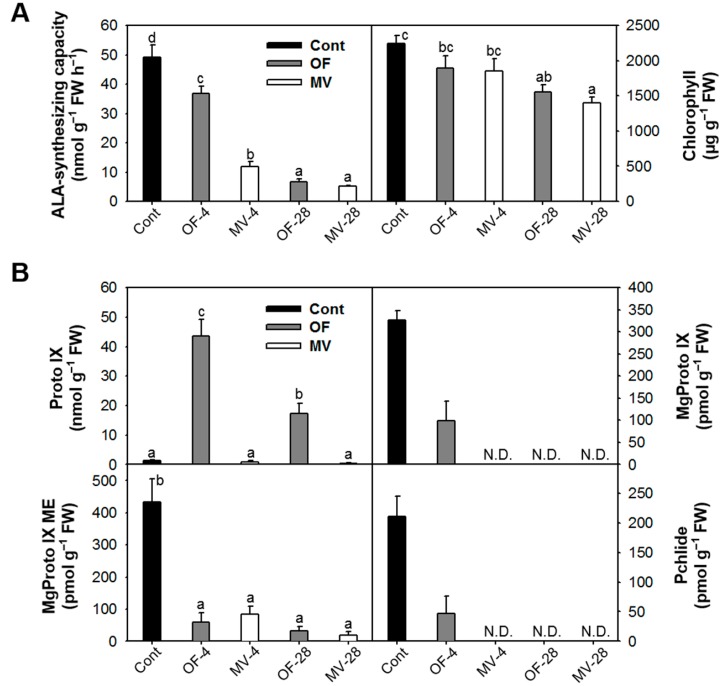
Changes in intermediates of porphyrin biosynthetic pathway in rice plants with the foliar application of OF or MV. (**A**) 5-aminolevulinic acid (ALA)-synthesizing capacity and chlorophyll content; and (**B**) Protoporphyrin IX (Proto IX) and Mg-porphyrin intermediates. The plants were subjected to the same treatments as in Figure 2. Treatment notations are the same as in Figure 2. N.D., not detected. The data represent the mean ± SE of six replicates from two independent experiments. Means denoted by the same letter did not differ significantly at *p* < 0.05 according to LSD test. Different letters indicate significant difference in statistics.

**Figure 6:**
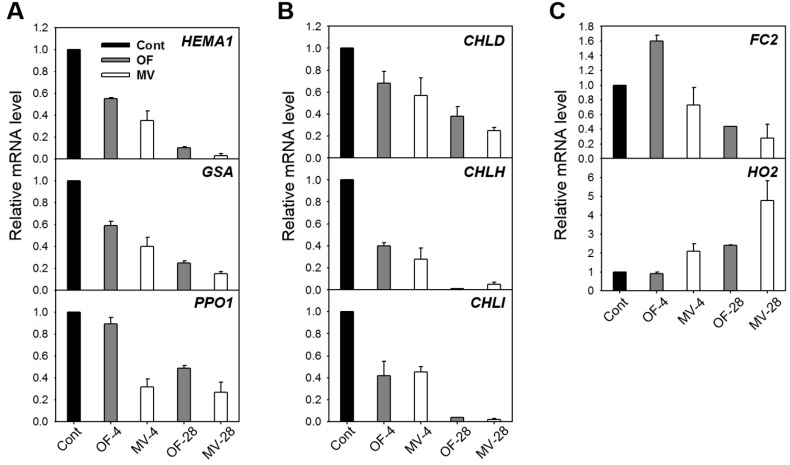
Expression of genes encoding the porphyrin pathway enzymes in rice plants with the foliar application of MV. (**A**) Common branch; (**B**) Mg-porphyrin branch; and (**C**) Fe-porphyrin branch. The plants were subjected to the same treatments as in Figure 2. Treatment notations are the same as in Figure 2. Total RNAs were purified from plants and reverse transcribed. The resultant cDNAs were used as templates for qRT-PCR using *Actin* as an internal control. The control 1 was used for normalization, with the expression level of the sample set to 1. Error bars represent SE, and representative data from three independent experiments are presented.

Many porphyrin-containing compounds are extremely harmful molecules as they produce powerful radicals such as ^1^O_2_ in the presence of light [1,4,27]. We examined the molecular mechanism of deregulated porphyrin biosynthesis in rice plants under photooxidative stress caused by OF or MV treatment. ALA formation, which is the rate-limiting step for porphyrin biosynthesis in light-grown plants [1,2], slightly decreased after 4 h of OF treatment and further decreased 28 h after the treatment (Figure 5A). Plants treated with MV drastically decreased ALA-synthesizing capacity after 4 h of the the treatment. We also assayed for the expression of genes involved in the synthesis of ALA, *HEMA1* encoding glutamyl-tRNA reductase and Glutamate 1-Semialdehyde Aminotransferase (*GSA*). Under photooxidative stress, transcript levels of *HEMA1* and *GSA* diminished gradually during OF and MV treatments, with a greater decline in MV-treated plants (Figure 6A). Content of chlorophyll, end product of Mg-porphyrin branch, slightly decreased in response to OF and MV treatments (Figure 5A). Proto IX prominently accumulated in OF-treated plants, with the highest level after 4 h of the treatment (Figure 5B). By contrast, transcript levels of *PPO1* encoding the enzyme which produces Proto IX decreased after 28 h of OF treatment (Figure 5B and Figure 6A). These results indicate that the accumulation of Proto IX results from accumulated Protogen IX which diffuses to the cytoplasm and is oxidized to Proto IX via peroxidase-like enzymes in plasma membrane as reported previously [5,6], but not from transcriptional regulation of *PPO*. The brown necrosis in OF-treated plants derived from the effect of peroxidation due to the great accumulation of Proto IX. On the other hand, Proto IX almost disappeared after 28 h of MV treatment, which may be due to photodynamic degradation of porphyrins.

Plants responded to OF treatment by greatly decreasing Mg-porphyrins including Mg-protoporphyrin IX (MgProto IX), MgProto IX methyl ester (ME), and Pchlide to levels lower than untreated control, with no detection of Mg-Proto IX and Pchlide after 28 h of the treatment, which derived from the inhibited conversion of Protogen IX to Proto IX and its subsequent steps (Figure 5B). In MV-treated plants, MgProto IX and Pchlide completely disappeared after 4 h of the treatment. In the Mg-porphyrin branch, the genes encoding the three Mg-chelatase subunits CHLH, CHLI, and CHLD were gradually down-regulated in OF- and MV-treated plants (Figure 6B). During the early stage of MV treatment, the decline of Mg-porphyrins occurred faster than the down-regulation of their biosynthetic genes, which can be largely attributed to photodynamic degradation rather than decreased levels of their biosynthesis. The latter may partly contribute to decreased levels of porphyrin intermediates. If excited porphyrins are left unquenched, they can form highly toxic radicals [28] and may endanger the plant cell. Therefore, a controlled flow of metabolites in the porphyrin biosynthetic pathway is essential to avoid photodynamic damage in stressed plants. Plants suffer severe photodynamic damage if these control mechanisms are circumvented, for example in plants treated with porphyrin deregulators including ALA and peroxidizing herbicides [29,30], plants with deregulation of porphyrin biosynthetic genes [31,32] or plants under environmental stress conditions [10,13]. The differential oxidative stress responses in OF- and MV-treated plants appear to be partly due to different perturbation of porphyrin metabolism, *i.e.*, excess accumulation of Proto IX and fast degradation of Mg-porphyrins, respectively.

In the Fe-porphyrin branch, transcript levels of *FC2*, which encode the plastidic isoform of Fe-chelatase, were up-regulated after 4 h of OF treatment but decreased after 28 h of the treatment, while MV-treated plants decreased transcript levels of *FC2* (Figure 6C). The increased expression of *FC*, which plays an essential role in heme synthesis, ensures the supply of cofactors for hemoproteins that may be required in response to OF-induced photooxidative stress and other oxidative stress conditions [33,34,35]. Transcript levels of *HO2* encoding heme oxygenase (HO), which catalyzes the formation of biliverdin-IXα from heme [36], increased after 28 h of OF treatment, whereas they began to increase after 4 h of MV treatment and further increased after 28 h (Figure 6C). HO is associated with heme degradation [37] and suggested as the antioxidant machinery through the role of its product biliverdin IXα in preventing oxidative stress in animals [38] and plants [12,39]. Our results demonstrate that porphyrin metabolism is tightly regulated via up-regulation of *HO2*, which could possibly be part of an efficient antioxidant response to photooxidative stress caused by OF and MV.

## 3. Experimental Section

### 3.1. Plant Growth and Pro-Oxidant Treatment

Germinated seeds of rice plants (*Oryza sativa* cv. Dongjin) were sown in pots filled with commercial greenhouse compost and were grown for three weeks in a greenhouse at 28 to 30 °C. Three days before commercial oxyfluorfen (OF, Goal) or methyl viologen (MV, *N*,*N*′-dimethyl-4,4′-bipyridinium dichloride) treatment, they were transferred to a growth chamber maintained at day/night temperatures of 28/25 °C under a 14-h-light/10-h-dark cycle with a 200 µmol·m^−2^·s^−1^ photosynthetic photon flux density. For the foliar application, three-week-old plants were sprayed with 50 µM OF or MV which develops similar degree of dehydration in the treated plants, placed in darkness for 1 h for allow absorbance, and then exposed to light (14-h day/10-h night) for two days. Control plants were treated with solvent only (30% acetone and 0.01% Tween 20). Parts of the youngest, fully developed leaves from control, OF- and MV-treated plants were sampled 4 and 28 h after the treatment. Technical-grade OF (Gyungnong, Gyeongju, Korea) was used for cellular leakage measurement.

### 3.2. Cellular Leakage

The rice leaf tissues were treated with OF and MV as described previously by Lee *et al.* [40] by cutting 4-mm leaf squares (0.1 g FW) with a razor blade and then placing them in a 6-cm diameter polystyrene Petri dish containing 5 mL of 1% sucrose and 1 mM MES (pH 6.5) with or without OF and MV. The tissues were incubated with various concentrations of OF and MV in a growth chamber at 25 °C in darkness for 12 h, and then exposed to continuous white light at 250 μmol·m^−2^·s^−1^ PPFD for 24 h. Cellular leakage was determined periodically by the detection of electrolyte leakage into the bathing medium using a conductivity meter (Cole-Parmer Instruments, Vernon Hills, IL, USA) as described by Lee *et al.* [40].

### 3.3. Determination of MDA Content

Lipid peroxidation was estimated by MDA content using a slight modification of the thiobarbituric acid (TBA) method described by Buege and Aust [41]. The treated leaf tissues (0.1 g) were homogenized with a mortar and pestle in 5 mL of a solution of 0.5% (*v*/*v*) TBA in 20% trichloroacetic acid (TCA). The homogenates were centrifuged at 20,000× *g* for 15 min, and the supernatants were collected. The supernatants were heated in a boiling water bath for 25 min then cooled in an ice bath. Following centrifugation at 20,000× *g* for 15 min, the resulting supernatants were used for spectrophotometric determination of MDA.

### 3.4. In Vivo Detection of H_2_O_2_

H_2_O_2_ was visually detected in the leaves using DAB [23]. The leaves were cut with a razor blade and incubated in a 1 mg·mL^−1^ solution of DAB (pH 3.8) for 4 h in light at 25 °C. The experiment was terminated by boiling the leaves in ethanol for 10 min. This treatment decolorized the leaves with the exception of the deep-brown polymerization product produced by the reaction of DAB with H_2_O_2_.

### 3.5. Measurement of Photosynthetic Activity

Chlorophyll *a* fluorescence was measured *in vivo* using a pulse amplitude modulation fluorometer (Handy PEA; Hansatech Instruments, Norfolk, UK) after dark adaptation for 20 min. The minimal fluorescence yield, F_o_, was obtained upon excitation with a weak measuring beam from a pulse light-emitting diode. The maximal fluorescence yield, F_m_, was determined after exposure to a saturating pulse of white light to close all reaction centers. The ratio of F_v_ to F_m_, representing the activity of PSII, was used to assess the functional damage to the plants.

### 3.6. Assays for Antioxidant Enzymes

Leaves (0.25 g) were ground to fine powder in a mortar under liquid N_2_. Soluble proteins were extracted by homogenizing the powder in 2 mL of 100 mM potassium phosphate buffer, pH 7.5, containing 2 mM EDTA, 1% PVP-40, and 1 mM phenylmethylsulfonyl fluoride. Equal amounts of protein were electrophoresed on 10% nondenaturing polyacrylamide gels at 4 °C for 1.5 h at a constant current of 30 mA. For the APX activity, gels were soaked in 50 mM potassium phosphate buffer, pH 7.0, containing 2 mM ascorbate for 30 min and stained as described in Rao *et al.* [42]. The CAT activity was detected by incubating the gels in 3.27 mM H_2_O_2_ for 25 min and staining them in a solution of 1% potassium ferricyanide and 1% ferric chloride for 4 min [43]. The staining of POD isozymes was achieved by incubating gels in sodium citrate buffer (pH 5.0) containing 9.25 mM *p*-phenylenediamine and 3.92 mM H_2_O_2_ for 15 min [44]. Gels were stained for SOD isoforms by soaking in 50 mM potassium phosphate (pH 7.8) containing 2.5 mM nitroblue tetrazolium (NBT) in darkness for 25 min, followed by soaking in 50 mM potassium phosphate (pH 7.8) containing 28 mM NBT and 28 μM riboflavin in darkness for 30 min. The gels were then exposed to light for 30 min [42].

### 3.7. ALA-Synthesizing Capacity

ALA-synthesizing capacity was measured as described by Papenbrock *et al.* [45]. Leaf disks were incubated in 20 mM phosphate buffer (pH 6.9) containing 40 mM levulinic acid in the light for 6 h. Samples were homogenized, resuspended in 1 mL of 20 mM phosphate buffer (pH 6.9), and centrifuged at 10,000× *g*. The 500-µL supernatant was mixed with 100 µL ethylacetoacetate, boiled for 10 min, and cooled for 5 min. An equal volume of modified Ehrlichs reagent was added and the absorption of the chromophore was determined at 553 nm.

### 3.8. Porphyrin Extraction and Analysis

Porphyrins were extracted and analyzed following the method of Lermontova and Grimm [46]. Leaf tissue was ground in methanol:acetone:0.1 N NaOH (9:10:1, (*v*/*v*)) and the homogenate was centrifuged at 10,000× *g* for 10 min. Porphyrin was separated by HPLC using a Novapak C_18_ column (4-µm particle size, 4.6 × 250 mm, Waters, Milford, MA, USA) at a flow rate of 1 mL·min^−1^. Porphyrins were eluted with a solvent system of 0.1 M ammonium phosphate (pH 5.8) and methanol. The column eluate was monitored using a fluorescence detector (2474, Waters) at excitation and emission wavelengths of 400 and 630 nm for Proto IX, 440 and 630 nm for Pchlide, and 415 and 595 nm for MgProto IX and MgProto IX ME, respectively. All porphyrins were identified and quantified using authentic standards. The chlorophyll content was spectrophotometrically determined according to the method of Lichtenthaler [47].

### 3.9. RNA Extraction and qRT-PCR

Total RNA was prepared from leaf tissues using TRIZOL Reagent (Invitrogen, Waltham, MA, USA), and 5 µg of RNA from each sample was used for the reverse transcription reaction (SuperScript III First-Strand Synthesis System, Invitrogen). Subsequently, 50 ng of cDNA was used for qRT-PCR analysis. The qRT-PCR analysis was carried out with the 7300 Real-Time PCR system (Applied Biosystems, Waltham, MA, USA) using Power SYBR Green PCR Master Mix (Applied Biosystems, Waltham, MA, USA) and specific primers for genes (Supplementary Table S1). The qRT-PCR program consisted of 2 min at 50 °C, 10 min at 95 °C, and 40 cycles of 15 s at 95 °C and 1 min at 60 °C. A melting curve analysis was performed after every PCR reaction to confirm the accuracy of each amplified product. All reactions were set up in triplicate. The control sample was used as the calibrator, with the expression level of the sample set to 1. *Actin* was used as the internal control.

## 4. Conclusions

The inhibition of porphyrin biosynthesis and PET by OF and MV, respectively, resulted in differential oxidative stress responses accompanied by not only greater increases in conductivity and MDA but also a greater decline of F_v_/F_m_ in MV-treated plants. During the late stage of photooxidative stress, the increases in SOD and CAT activities, as well as *SODA* and *CATA* transcripts, were greater in MV-treated plants than in OF-treated plants, whereas the increases in APX activity and its transcript levels were greater in OF-treated plants. Porphyrin metabolism also could provide antioxidant machinery via up-regulation of *HO2* in OF- and MV-treated plants. These efficient antioxidative defense systems may partially detoxify certain levels of photosensitizing pophyrin products and ROS but were not sufficient to overcome photooxidative damage.

Both OF- and MV-treated plants exhibited a substantial down-regulation of *HEMA1*, *PPO1*, and the genes involving Mg-porphyrin synthesis as well as almost complete disappearance of Mg-porphyrins during the late stage of photooxidative stress. Particularly, the plants treated with MV rapidly scavenged toxic porphyrin intermediates mainly through photodynamic degradation rather than down-regulation of most genes in porphyrin biosynthesis. We have shown that the decline in phytotoxic porphyrin intermediates is important to avoid porphyrin-mediated photodynamic damage under excess ROS levels derived from perturbations of porphyrin biosynthesis or PET. Not only increased antioxidant responses, *i.e.*, ROS-scavenging enzymes and *HO*2, but also rapid scavenging of photosensitizing porphyrins are suggested as cooperating mechanisms for preventing ROS-induced damage under the photooxidative stress generated by OF or MV.

## Supplementary Materials

Supplementary materials can be found at http://www.mdpi.com/1422-0067/16/07/16529/s1.
