# Supplementary material for: Differential Antioxidant Responses and Perturbed Porphyrin Biosynthesis after Exposure to Oxyfluorfen and Methyl Viologen in Oryza sativa
Source: Int J Mol Sci. 2015 Jul 21;16(7):16529–44. doi: 10.3390/ijms160716529 (PMC4519964; doi:10.3390/ijms160716529)
Supplement: Supplementary file 1 [file ijms-16-16529-s001.pdf]

Supplementary Information

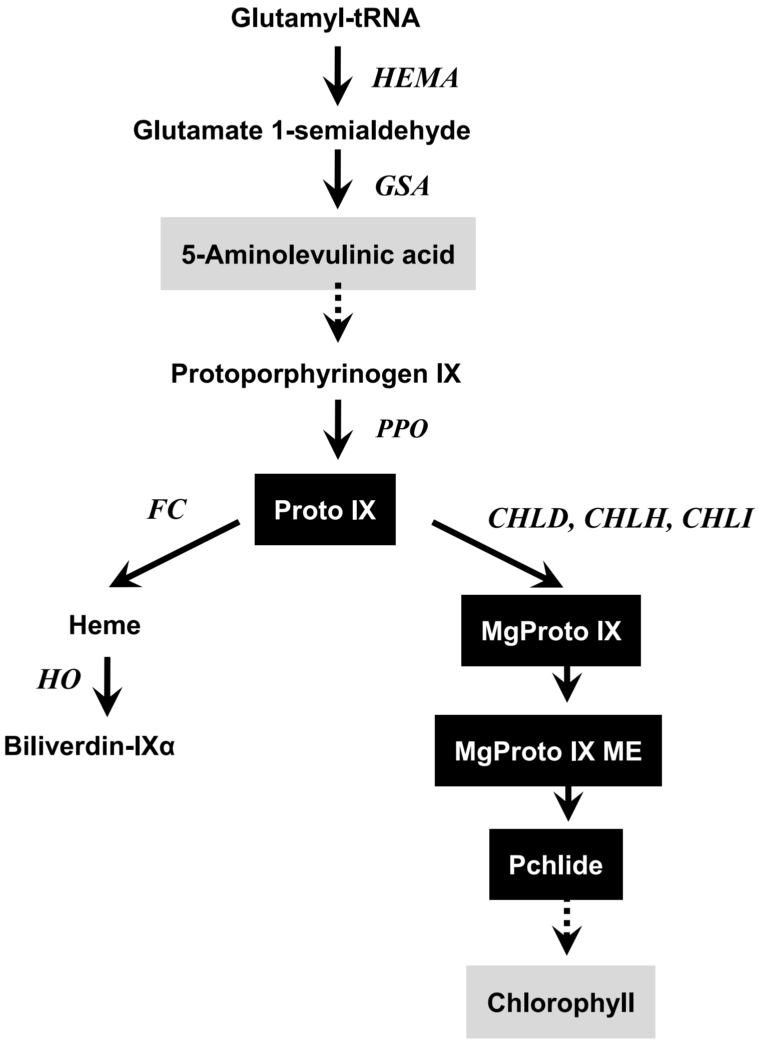

Figure S1. The porphyrin biosynthetic pathway in plants showing intermediates and genes analyzed in this study. Intermediates quantified in this study are highlighted. Intermediates: Proto IX, protoporphyrin IX; MgProto IX, Mg-protoporphyrin IX; MgProto IX ME, Mg-protoporphyrin IX methyl ester; Pchlide, protochlorophyllide. Genes and enzymes that correspond to the gene names: *HEMA*, glutamyl-tRNA reductase; *GSA*, glutamate 1-semialdehyde aminotransferase; *PPO*, protoporphyrinogen oxidase; *FC*, Fe-chelatase; *HO*, heme oxygenase; *CHLD*, D-subunit of Mg-chelatase; *CHLH*, H-subunit of Mg-chelatase; *CHLI*, I-subunit of Mg-chelatase.

Table S1. Primers used for qRT-PCR assays.

Gene	Primer Sequence
HEMA1	F: GCTATGGGTGGTGTTCGACT<br>R: CGATCTTCTGGAGGCACTTC
GSA	F: CTCCGTGACTTGACGAAACA<br>R: GTAGGTTCCAGGCTCCATCA
PPO1	F: ACAGTTCCTCATTGGCCATC<br>R: CCCATGAAATTTTTGCTGCT
CHLD	F: TGGGACAGCAAAGACAGTGA<br>R: AAGGCCAGGTTGAAACACAG
CHLH	F: GTGTGGGTTGCGTTCTTTTT<br>R: GGTGACAATGTGGCTCCTCT
CHLI	F: TGTGCTTCTGGATTCTGCTG<br>R: GCTGGAGCTTGTCTTGTTCC
FC2	F: TTGGTGCTATGGCAGTTTCA<br>R: AGTGGAACAAAGGCAGGATG
HO2	F: AGGGACCTAGCAGCCCTAAC<br>R: CCCGTATCGTCCATCTTGAG
SODA	F: ACCTTCCATCTCCCAGCAC<br>R: ACAAATGGTACCCTTCAGCTT
APXA	F: ACAAAGCCCTGCTGAGTGAC<br>R: TAACAGCCCACCGAGACATT
APXB	F: CCAAGTGACAAAGCCCTCAT<br>R: TCTTGACAGCAAATAGCTTGG
CATA	F: CCAGTGTGATGAGTCGTTGG<br>R: ATATGCAGGCTCCATTTTGG
CATB	F: AAGGATGGAAAGCCTCACCT<br>R: AGGGGGATGATATCCTCTGG
CATC	F: ACAACCACTACGACGGCTT<br>R: CCAGTAGGAGAGCCAGATGC
ACTIN	F: CTTCATAGGAATGGAAGCTGCGGGTA<br>R: CGACCACCTTGATCTTCATGCTGCTA

F, forward; R, reverse.
